# Parliament and the Profession

**Published:** 1918-01-26

**Authors:** 


### Parliament and the Profession.

### National Service Medical Boards.

IN reply to a question put by Mr. Snowden to the Under-Secretary of State for War, it was stated by Mr. Beck, Parliamentary Secretary to the Ministry of National Service, that all members of the National Service medical boards are civilian medical practitioners. Certain of the presidents of the recruiting medical boards continued to act as chairmen of the National Service medical boards during the period of transference of the medical department from the control of the Army Council to that of the Ministry of National Service, but these have now been relieved with the exception of two, who will be relieved within a few days.

### Midwives (Ireland) Bill.

MR. DUKE informed General M'Calmont that certain provisions of the Midwives (Ireland) Bill are not yet agreed upon with the parties concerned. Unless agreement is arrived at in the course of the week, he thought it would not be practicable to proceed with the Bill before the Prorogation. A question by Sir J. D. Rees, asking if this decision could be reconsidered in view of the fact that the services of these ladies would be required at the birth of the new Constitution, did not elicit a reply.

### Army Medical Service.

MR. MACPHERSON informed Major Davies that it is not intended to place the Director-General of Army Medical Services on the Army Council, but whenever matters affecting medical organisation or administration are before the Council, the Director-General will be called on to give the Council the benefit of his advice.

### Anthropometers.

IN a question to the Minister of Munitions, Mr. Jowett suggested that the manufacture of anthropometers (instruments designed by Madame Montessori for use in Montessori schools for weighing and measuring children) was being impeded owing to the nature of the material necessary for manufacture. Sir W. Evans said that there is no prohibition, and any applications for such instruments would be carefully and sympathetically considered.

